# Mitigation of CSI Temporal Phase Rotation with B2B Calibration Method for Fine-Grained Motion Detection Analysis on Commodity Wi-Fi Devices

**DOI:** 10.3390/s18113795

**Published:** 2018-11-06

**Authors:** Nopphon Keerativoranan, Azril Haniz, Kentaro Saito, Jun-ichi Takada

**Affiliations:** School of Environment and Society, Tokyo Institute of Technology, Tokyo 152-8552, Japan; azril@ap.ide.titech.ac.jp (A.H.); saitouken@tse.ens.titech.ac.jp (K.S.); takada@ide.titech.ac.jp (J.-i.T.)

**Keywords:** Wi-FI, channel state information, motion sensing, device-free, phase calibration, wavelet transform

## Abstract

Limitations of optical devices for motion sensing such as small coverage, sensitivity to obstacles, and privacy exposure result in the need for improvement. As motion sensing based on radio frequency signals is not constrained by the limitation above, channel state information (CSI) from Wi-Fi devices could be used to improve sensing performance under the above circumstances. Unfortunately, CSI phase cannot be practically obtained due to the temporal phase rotation generated from Wi-Fi chips. Therefore, it would be rather complicated to realize motion analysis, especially the direction of motion. To mitigate the issue, this paper proposes a CSI calibration method that employs a back-to-back channel between Wi-Fi transceivers for phase rotation removal while preserving the original CSI phase. Through experiment, calibrated CSI showed a high similarity to the channel without phase rotation measured using a Vector Network Analyzer (VNA). Another experiment was conducted to observe Doppler frequency due to simple hand gestures using the Wavelet transform. A visual analysis revealed that the Doppler frequency of calibrated CSI could correctly capture the motion pattern. To the best of the authors’ knowledge, this is the first calibration method that maintains the original CSI and is applicable for in-depth motion analysis.

## 1. Introduction

Currently, technology in human-computer interaction (HCI) is moving toward the contactless interface where users could communicate with any computing devices by merely performing a particular gesture in the air. Some vision-based motion sensing systems have already been commercialized such as Kinect, Leap Motion, and Orbbec. Although these systems promise high tracking accuracy and precision, it unavoidably has to deal with the physical limitation of the optical device. For example, it has small active sensing area which highly depends on the focal length of the lens, operates only in the presence of line-of-sight (LoS) between device and user, and might raise a concern in privacy exposure issue [1,2,3]. These constraints may be a few of the reasons why commercial virtual reality (VR) and augmented reality (AR) handsets such as gear VR controller, data glove, or smartphone require a wearable device for interaction. However, hand-equipped sensors are intrusive and inconvenient, and may not be practical for specific applications such as elderly care, and intrusion detection [3,4]. To overcome these limitations, an alternative motion sensing approach which is device-free, ubiquitous, and low-cost could be a good candidate in assisting or substituting the current system. Leveraging commercial radio frequency (RF) system (Wi-Fi, Bluetooth, RFID, etc.) is one solution because RF is not affected by these limitations. In fact, RF received signal strength indicator (RSSI) based motion sensing has been researched comprehensively in [5,6,7,8]. Unfortunately, the nature of RSSI is highly interference-sensitive, device-dependent, and easily affected by external conditions such as temperature and humidity [9,10,11]. Therefore only simple gesture recognition would be achievable rather than performing tracking.

**Figure 1:**
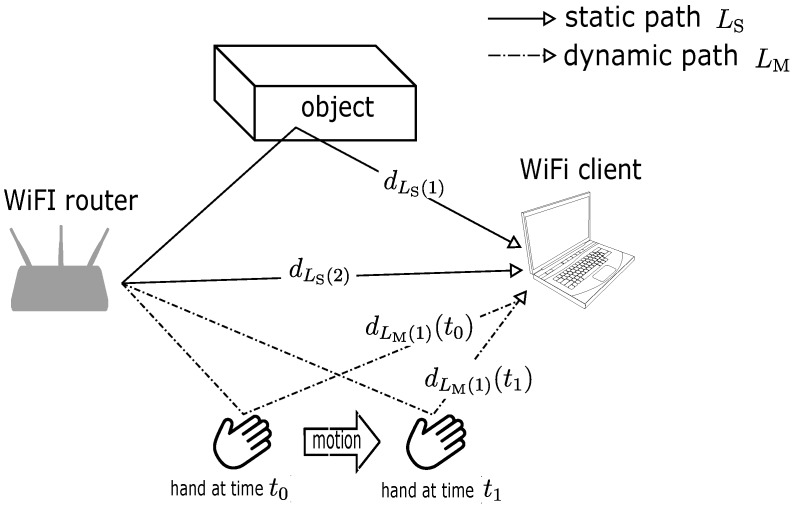
Propagation of Wi-Fi signal in multipath channel interacting with static object and hand in motion.

With respect to recent works in the extraction of channel state information (CSI) from the physical layer of commodity Wi-Fi network interface cards (NIC) [12,13], which arguably contains a lot more information than RSSI, fine-grained motion sensing and indoor localization with Wi-Fi devices have gained much attention from researchers, and various works using CSI have increased drastically [14,15,16,17,18,19,20,21,22]. Generally, Wi-Fi chips estimate CSI which is represented by the complex channel frequency transfer function of the wireless channel between two Wi-Fi transceivers at each orthogonal frequency division multiplexing (OFDM) subcarrier. In the simple transmission depicted in Figure 1, the Wi-Fi signal propagates through a wireless channel on different paths, and the superposition of multipath signals at receiver is translated onto CSI as channel fading, and it supposedly remains unchanged with the same configuration and environment. However, in the presence of motion, a certain path which interacts with the moving object will experience changes with time. This effect could be observed in the CSI as the rotation of phase components and a time-varying channel fading in the amplitude component. The time-varying CSI will result in a peak in the Doppler spectrum which is directly proportional to the object’s velocity in the direction concerning a link between Wi-Fi transceivers. In actual measurements, unfortunately, the temporal frequency offset of local oscillators (LOs) between two Wi-Fi transceivers due to the absence of synchronization obscures the CSI phase component, and therefore phase cannot be practically used. As the frequency offset is manifested at the CSI phase as an additional phase rotation, the term phase rotation will be used to describe this effect throughout the paper although it is widely referred to as the phase offset in previous works.

As a consequence, performing any motion analysis is infeasible without separation of the phase rotation from the CSI. Besides, spatial mapping (SM) and cyclic shift diversity (CSD) modulated in the transmitter may remain attached to the extracted CSI when operating in multiple transmit streams configuration [23,24]. Hence, the typical CSI model could not be applied as it does not purely represent the specific channel and regrettably limits the applicability of the system to only pattern recognition-based motion analysis. To fully use CSI for motion analysis, phase rotation calibration without affecting the original CSI phase is needed. Many works on CSI-based movement detection and recognition system have proposed various techniques for dealing with the phase rotation during data preprocessing. Using CSI amplitude approach has been widely used to avoid the effect of phase rotation. However, without the CSI phase component, only a relative magnitude of speed could be obtained. Therefore this approach is only suitable for analyzing motion with a periodic profile such as breathing detection [25,26], or predefined unique pattern such as human gestures [18,19,20,27] and human activities detection [14,16,17,28]. The phase sanitization approach compensates for the phase rotation by linearly removing the mean and slope of the measured CSI phase. This is a double-edged approach in which phase rotation is mitigated but simultaneously a part of the CSI is proportionally removed along with it. Although the resulting sanitized phase has been implemented in many works [15,22,29,30], to the best of our knowledge, there is no justification of what the sanitized phase physically represents. Therefore the application is quite limited and mostly case-dependent. The phase difference method, applied in [2,21,31,32], mitigates the phase rotation by using the CSI phase measured from another receive antenna. Since the phase rotation between both antennas was generated by the same Wi-Fi chip, it should be canceled out after subtraction. Although this method could remove the phase rotation, the result is still not yet the original CSI phase but a phase difference between two antennas. Even though various phase rotation mitigation techniques have been implemented in previous works, it is impossible to remove the phase rotation without sacrificing partial or entire phase information.

In order to effectively suppress the phase rotation without contaminating the CSI, we introduce a calibration method by using a back-to-back (b2b) channel in this paper. Furthermore, this technique also can remove SM and CSD matrices without requiring their knowledge in the process and thus returning the channel matrix without distortion. For validation, the experiment was performed to compare between the calibrated CSI with the channel measured using a Vector Network Analyzer (VNA) as ground truth. The results have successfully shown a similarity of the CSI phase component relative to the ground truth albeit with the constant residual phase offset. After removing the constant residual offset, the CSI phase closely resembled the result from the ground truth with a root-mean-square error (RMSE) of approximately 0.117 radians. Another experiment was conducted to observe the Doppler frequency of hand-waving gesture using the calibrated CSI. A bell-shaped profile due to the gesture was observed as the higher power spectrum in the time-Doppler domain. Furthermore, the relative motion direction was also correctly detected from the sign of Doppler frequency according to the bistatic Doppler model [33]. We believe that our CSI calibration method provides a solution for recovering Wi-Fi CSI without phase rotation in practice.

The rest of the paper is organized as follows: the basic concept of channel state information and the time-varying phase rotation model are addressed in Section 2. Then our CSI calibration model is elaborately detailed in Section 3 followed by the experimental results and discussion in Section 4. Finally, the findings of this report are summarized in Section 5.

## 2. Preliminaries and CSI Phase Rotation

### 2.1. Channel State Information

Multiple-input multiple-output (MIMO) transmission and beamforming have been introduced in many wireless communication systems in order to improve data transmission rate and reliability. They require a knowledge of real-time CSI to perform spatial multiplexing and diversity techniques. Therefore, a CSI frame has been included in the physical layer (PHY) of Wi-Fi systems since the IEEE 802.11n standard, specifically training symbols in the high throughput long training field (HT-LTF) preamble format [23]. Wi-Fi uses the OFDM modulation scheme for transmission thus signal is transmitted through $N_{K}$ subcarriers simultaneously. Let us define $X_{n_{T}}\left(t,f_{k}\right)$ and $Y_{n_{R}}\left(t,f_{k}\right)$ as the known transmitted and received HT-LTF training symbols in frequency domain measured at time *t*. The wireless channel at each subcarrier frequency $f_{k}$ can be estimated in terms of the CSI matrix $H_{n_{R},n_{T}}\left(t,f_{k}\right)$, where *k* represents subcarrier frequency index ranging from −28 to 28 corresponding to $N_{K}=56$ subcarrier frequencies in 20 MHz bandwidth and −58 to 58 for 114 subcarrier frequencies in 40 MHz bandwidth. This channel matrix corresponds to the link between $n_{T}$-th transmit antenna and $n_{R}$-th receive antenna, and has a dimension of $N_{T}\times N_{R}\times N_{K}$ where $N_{T}$ and $N_{R}$ are the number of transmit and receive antennas. The estimated CSI matrix can be expressed by

(1) $$Y_{n_{R}}\left(t,f_{k}\right)=H_{n_{R},n_{T}}\left(t,f_{k}\right)X_{n_{T}}\left(t,f_{k}\right)+Z$$

assuming the channel has an additive white Gaussian noise *Z*. In a nutshell, the CSI is physically a collection of Wi-Fi OFDM signals getting attenuated, faded, and scattered by interacting objects (IOs) during propagation which can be modeled as,

(2) $$H_{n_{R},n_{T}}\left(t,f_{k}\right)=\sum_{l\in L}\alpha_{l}\left(f_{k},t\right)e^{\frac{-j2\pi d_{l}\left(t\right)}{\lambda_{k}}}$$

where $\lambda_{k}$, $\alpha_{l}$, and *L* are wavelength at subcarrier frequency index *k*, complex attenuation for *l*-th path, and a set of paths which signal propagate with the length of $d_{l}\left(t\right)$ respectively. The component of CSI corresponding to the signal propagated through the path length $d_{l}\left(t\right)$ should remain unchanged if the *l*-th path signal only interacts with stationary IOs. However, the interaction with the moving IOs will cause the variation of complex attenuation $\alpha_{l}\left(f_{k},t\right)$ and the change of path length $d_{l}\left(t\right)$ in terms of Doppler frequency shift [34] which will be explained later in Section 3.2. The measured channel ${\hat{H}}_{n_{R},n_{T}}\left(t,f_{k}\right)$ is contaminated by a phase rotation term generated from the non-synchonizied LOs of both Wi-Fi transceivers and will be discussed in Section 2.3. The model can be extended from Equation (2) into a summation of the dynamic component ${\hat{H}}_{n_{R},n_{T}}^{M}\left(t,f_{k}\right)$, and the static component ${\hat{H}}_{n_{R},n_{T}}^{S}\left(f_{k}\right)$, and a muliplication with the time-varying phase rotation term $e^{-j\phi (t,k)}$ [14,35] as shown below.

(3) $${\hat{H}}_{n_{R},n_{T}}\left(t,f_{k}\right)=\left[{\hat{H}}_{n_{R},n_{T}}^{M}\left(t,f_{k}\right)+{\hat{H}}_{n_{R},n_{T}}^{S}\left(f_{k}\right)\right]e^{-j\phi (t,k)}+Z$$

where

(3a) $$\begin{array}{c} {\hat{H}}_{n_{R},n_{T}}^{M}\left(t,f_{k}\right)=\sum_{m\in L_{M}}\alpha_{m}\left(t,f_{k}\right)e^{\frac{-j2\pi d_{m}\left(t\right)}{\lambda_{k}}} \end{array}$$

(3b) $$\begin{array}{c} {\hat{H}}_{n_{R},n_{T}}^{S}\left(f_{k}\right)=\sum_{s\in L_{S}}\alpha_{s}\left(f_{k}\right)e^{\frac{-j2\pi d_{s}}{\lambda_{k}}} \end{array}$$

The dynamic component consists of a set of signals propagated via $L_{M}\subset L$ paths, interacting with moving IOs, and then causing CSI phase rotation due to a change of path length $d_{m}\left(t\right)$. On the other hand, signals travelling through a set of paths $L_{S}\subset L$ in the static component does not experience any change in path length $d_{s}$ because they all interact with stationary IOs thus keeping CSI constant as depicted in Figure 1.

### 2.2. Effect of Spatial Diversity and Effective CSI

**Figure 2:**
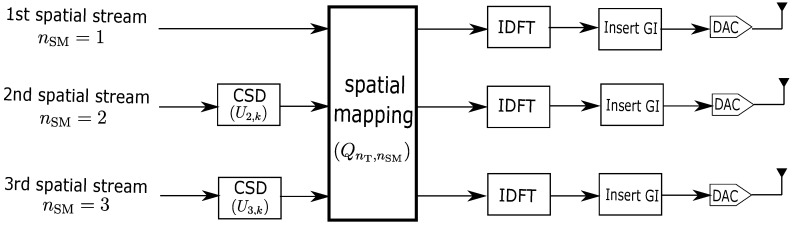
Partial Wi-Fi transmitter block diagram with 3 spatial streams being mapped by spatial mapping (SM) and cyclic shift diversity (CSD) according to IEEE 802.11n standard.

In the case of MIMO configuration, each OFDM symbol $X_{n_{T}}\left(t,f_{k}\right)$ is firstly allocated into multiple spatial streams, then being shifted and/or multiplied by the spatial matrix (SM) and cyclic shift diversity (CSD) before transmission according to the IEEE 802.11n standard [23] as shown in Figure 2. SM matrix $Q_{n_{T},n_{\mathrm{SM}}}$ where $n_{\mathrm{SM}}\in \left\{1,2,\ldots ,N_{\mathrm{SM}}\right\}$ modulates $N_{\mathrm{SM}}$ spatial streams into $N_{T}$ transmit antennas with one of the mapping techniques mentioned in the standard. For instance, direct mapping assigns a single spatial stream per antenna while indirect mapping mixes all spatial streams with different weights to each antenna. CSD, defined in the standard as a matrix $U_{n_{\mathrm{SM}},k}=e^{-j2\pi k\Delta_{F}T_{n_{\mathrm{SM}}}^{\mathrm{CSD}}}$, rotates the spatial streams with different delays $T_{n_{\mathrm{SM}}}^{\mathrm{CSD}}$ where $\Delta_{F}$ is the gap between consecutive subcarrier frequencies.

Regarding the IEEE 802.11n standard, there are two methods to obtain CSI depending on where CSI estimation is calculated. Considering a scenario where transmission is initiated at ST1 Wi-Fi transmitter which sends a signal $X_{n_{\mathrm{ST}1}}\left(t,f_{k}\right)Q_{n_{\mathrm{ST}1},n_{\mathrm{SM}}}U_{n_{\mathrm{SM}},k}$ to ST2 Wi-Fi receiver. In the implicit method, the estimation of channel ${\hat{H}}_{n_{\mathrm{ST}2},n_{\mathrm{ST}1}}\left(t,f_{k}\right)$ from ST1 to ST2 is calculated at transmitter side deriving from a reverse channel ${\hat{H}}_{n_{\mathrm{ST}1},n_{\mathrm{ST}2}}\left(t,f_{k}\right)$ after receiving a feedback signal from ST2. The other method is an explicit one where the channel is estimated directly at receiver side. However, this method in practice cannot separate the SM matrix from CSI because ST1 transmitter does not provide spatial mapping to ST2 receiver. Additionally, it may not have removed CSD matrix from CSI despite having information of CSD defined in Wi-Fi standard. This type of CSI is called effective CSI [23,24],

(4) $${\hat{H}}_{n_{\mathrm{ST}2},n_{\mathrm{ST}1}}^{\mathrm{eff}}\left(t,f_{k}\right)={\hat{H}}_{n_{\mathrm{ST}2},n_{\mathrm{ST}1}}\left(t,f_{k}\right)Q_{n_{\mathrm{ST}1},n_{\mathrm{SM}}}U_{n_{\mathrm{SM}},k}$$

For instance, an open-source Linux 802.11n CSI tool developed by [12] used the explicit method to capture the effective CSI per received Wi-Fi packet. Since the tool was applied in this work, our CSI calibration inevitably has to remove SM and CSD matrices aside from phase rotation.

### 2.3. CSI Phase Rotation

**Figure 3:**
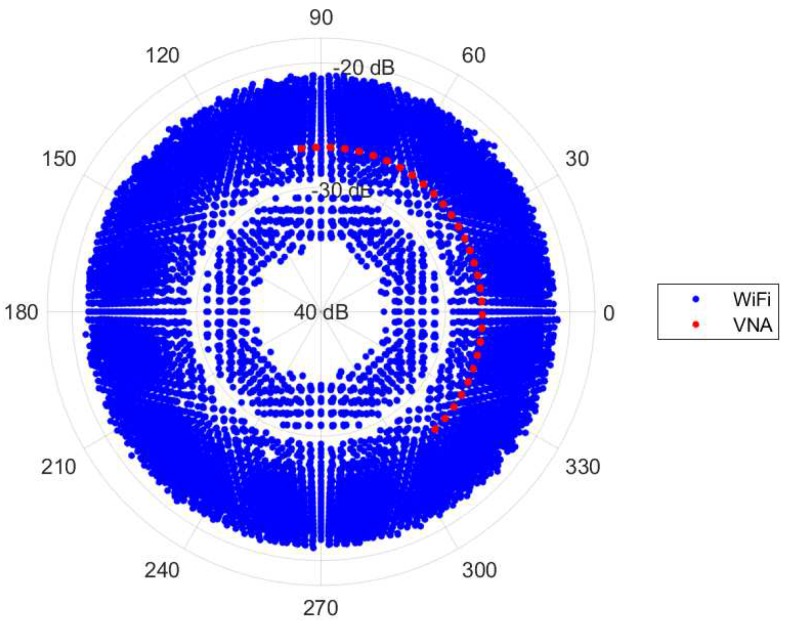
2000 snapshots of the phase of Wi-Fi channel state information (CSI), blue dots, and the phase of a channel measured by vector network analyzer (VNA), red dots, under the same b2b configuration plotted in polar coordinates.

The phase component of CSI is the most significant feature for motion detection analysis. This is because when there is any motion, the set of signal that interact with these dynamic IOs will physically experience change in their propagation paths. Specifically, $d_{m}\left(t\right)$ in Equation (3a) keeps changing with time and therefore exhibited as a phase rotation in the measured CSI, ${\hat{H}}_{n_{R},n_{T}}\left(t,f_{k}\right)$. Unfortunately, this phenomena could not be directly observed because it has been corrupted by the additional time-varying phase rotation ϕ(t,k) due to non-synchronized LOs. To visualize the effect of ϕ(t,k) on CSI phase, we connected two Wi-Fi stations with a cable and measured the back-to-back (b2b) channel of 30 subcarrier frequencies with 2000 snapshots every 0.4 ms. An attenuator was inserted in between to prevent damage to the Wi-Fi devices. In comparison, the channel without phase rotation was measured using a VNA with the same configuration. Figure 3 shows that Wi-Fi CSI phase (blue dot) appears to be spinning around the polar coordinate plane even in the static wired channel. In contrast, the channel measured using the VNA (red dot) has a relatively stable phase in all 30 frequencies with a constant phase shift between consecutive frequencies which seems to agree with Equation (3). Hence, it is a non-trivial task to mitigate the phase rotation term $e^{-j\phi (t,k)}$ in a measured Wi-Fi CSI before attempting any further analysis.

The sources of phase rotation have been widely discussed throughout CSI phase related literature. There are at least four sources listed below that contribute to CSI phase information.

1. **Carrier frequency offset (CFO)** [2,13,29,36,37,38,39] carrier frequency of the transmitter and receiver are not precisely matched because both are generated from non-synchronized LOs whose frequency offset are independently shifting and varying over time. Consequently, the phase of all CSI subcarriers will suffer from a temporal phase offset $\phi_{C}\left(t\right)$.
2. **Sampling frequency offset (SFO)** [2,13,22,29,36,38,39,40] since ADC clocks of the transmitter and receiver are not synchronized, both will drift separately. Therefore, each received signal will experience a time-varying delay offset with respect to the transmitter. In the frequency domain, the offset is represented in the CSI phase as an additional phase rotation $\phi_{S}\left(t\right)$ proportional to subcarrier index *k*.
3. **Packet boundary delay (PBD)** [2,13,35,36,37,39,40] after an OFDM symbol passes through the ADC, the receiver estimates the boundary of the OFDM symbol by using correlation detection. The estimated boundary may include a delay offset as long as orthogonality of the OFDM symbol is preserved, and its delay is less than the guard band interval. Similar to SFO, this delay exhibits in the frequency domain as another additional phase rotation $\phi_{P}\left(t\right)$ proportional to subcarrier index.
4. **Phase-locked loop offset (PLO)** [2,36,37] phase-locked loop circuit is in charge of generating a center frequency for both the transmitter and receiver. As both are using different chips, it will also individually create some initial random phase when the Wi-Fi NIC is initialized causing phase difference to the received OFDM symbol. As a result, CSI phase will be added with a relatively constant phase offset $\phi_{\mathrm{PLL}}$.

The time-varying phase rotation ϕ(t,k) can be modeled as a summation of these four sources as follows.

(5) $$\phi \left(t,k\right)=\left[\phi_{P}\left(t\right)+\phi_{S}\left(t\right)\right]k+\phi_{C}\left(t\right)+\phi_{\mathrm{PLL}}$$

**Figure 4:**
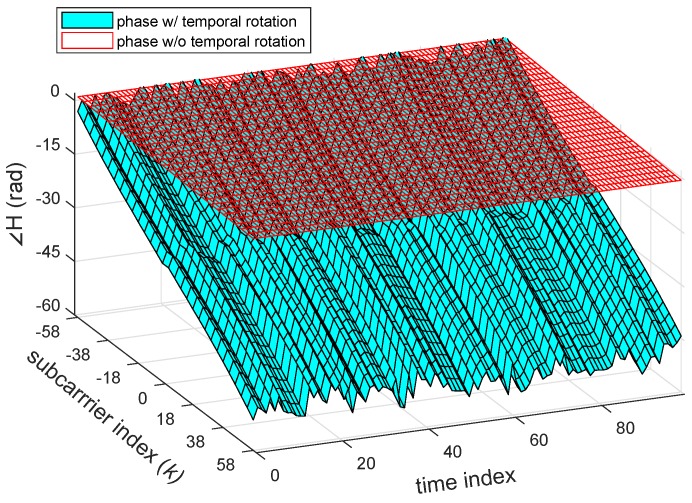
Effect of the temporal phase rotation to the CSI phase (cyan) in comparison with the original phase component (red).

Figure 4 visualizes the effect from the phase rotation’s sources causing the time-varying shift and rotation of the CSI phase away from the channel without the phase rotation. Unfortunately, even though a model of ϕ(t,k) can be formed, it is a challenging task to filter out the rotations because we cannot directly obtain their parameters from the NIC. Hence, other indirect signal processing techniques are needed for calibration of CSI phase rotation. It is worth noting that, although NIC has implemented CFO and SFO correction, it may not be able to remove these rotations entirely [29,36]. As clearly seen in Figure 3, the results after correction would still be the residual phase rotation added to CSI phase.

## 3. Phase Rotation Calibration for Motion Detection Analysis

### 3.1. CSI Calibration Model

The goal of our CSI calibration method is to virtually subdue phase rotation and maintain original CSI in both the amplitude and phase components. The calibration should be able to analytically explain the effect of movement in a target CSI which is assumed to be the measured channel ${\hat{H}}_{n_{R},n_{T}}\left(t,f_{k}\right)$. Since it is infeasible to precisely acquire the parameters of phase rotation from NIC, the practical solution to remove them would be the normalization of the target CSI with a reference CSI that suffers the same rotations. Hence, it is argued that the ideal reference CSI for phase rotation removal should have the following properties. It should be a static channel which is independent from the target CSI, and both experience the same phase rotation which can be written as

(6) $$H_{\mathrm{REF}}\left(t,f_{k}\right)=\left[\sum_{b\in L_{B}}\alpha_{b}\left(f_{k}\right)e^{\frac{-j2\pi d_{b}}{\lambda_{k}}}\right]e^{-j\phi (t,k)}$$

where $L_{B}$ is a set of path propagating in the reference channel and $L_{B}⊄L$. Subsequently, the calibrated CSI can be formulated as the CSI in Equation (3) being normalized by the transfer function of the reference channel,

(7) $${\tilde{H}}_{n_{R},n_{T}}\left(t,f_{k}\right)=\frac{{\hat{H}}_{n_{R},n_{T}}\left(t,f_{k}\right)}{H_{\mathrm{REF}}\left(t,f_{k}\right)}=\frac{\sum_{m\in L_{M}}\alpha_{m}\left(t,f_{k}\right)e^{\frac{-j2\pi d_{m}\left(t\right)}{\lambda_{k}}}+\sum_{s\in L_{S}}\alpha_{s}\left(f_{k}\right)e^{\frac{-j2\pi d_{s}}{\lambda_{k}}}}{\sum_{b\in L_{B}}\alpha_{b}\left(f_{k}\right)e^{\frac{-j2\pi d_{b}}{\lambda_{k}}}}+Z$$

This calibration, however, does not consider the effect of SM and CSD, so $Q_{n_{T},n_{\mathrm{SM}}}$ and $U_{n_{\mathrm{SM}},k}$ must be detached from the effective CSI in Equation (4). In fact, the effect of CSD can be negligible for motion analysis because it only adds a constant phase $\Delta T^{\mathrm{CSD}}$ to the calibrated CSI. On the other hand, SM must unavoidably be removed beforehand if the transmitter does not use direct spatial mapping. This is because the element $n_{T}$ of CSI does not represent the channel coming from single transmit stream but the mixture between multiple streams.

**Figure 5:**
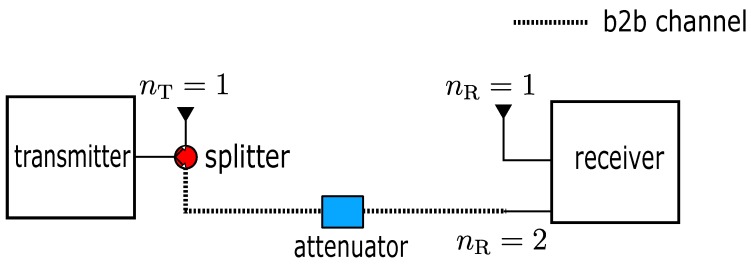
1×2 single-input multiple-output (SIMO) configuration describing the implementation of simplified CSI calibration technique in Equation (8).

Regarding the practical reference CSI for the implementation of our calibration method, the previous works of using CSI amplitude and the phase difference approaches also use the concept of reference CSI where the target CSI itself and the CSI measured from different antenna port are treated as the reference respectively. As it was mentioned in Section 1, although both methods could remove the phase rotation, the partial or entire CSI phase is also inevitably removed because these reference channels are not independent from the target CSI. The CSI measured from a transmit antenna and the receive antenna located far away relative to the location of moving IOs could satisfy the condition of the reference CSI. Another candidate for the reference CSI could be the wireless channel whose environment is shielded by the RF absorber, thus resulting in the static and independent channel. Although these configurations may be feasible for our calibration conditions, we decided to apply the CSI obtained through the b2b channel as reference CSI because not only it fits all criteria stated previously, the propagation path of signal is also limited to a single path through the cable ($b_{0}$-th path) and consequently simplifies the reference CSI Equation (6). Moreover, we are able to practically calculate a constant phase shift due to path length $d_{b_{0}}$ of the b2b channel. Assume the target CSI is measured between the first transmit antenna and the first receive antenna $\left(n_{T}=n_{R}=1\right)$. For the sake of simplification, this b2b connection is used to measure the channel corresponding to the channel coming from the same transmit antenna port but different receiver port $\left(n_{T}=1,n_{R}=2\right)$ as depicted in Figure 5. In case of the effective CSI in this configuration, both target and b2b channels will have the same SM $\left(Q_{1,n_{\mathrm{SM}}}\right)$ and CSD $\left(U_{n_{\mathrm{SM}},k}\right)$ of the first transmit antenna $\left(n_{T}=1\right)$, and thus will also be canceled out together with the phase rotation during calibration. Hence, Equation (7) can be simplified as below,

(8) $${\tilde{H}}_{n_{R},n_{T}}\left(t,f_{k}\right)=\sum_{m\in L_{M}}\frac{\alpha_{m}\left(t,f_{k}\right)}{\alpha_{b_{0}}\left(f_{k}\right)}e^{\frac{-j2\pi }{\lambda_{k}}\left(d_{m}\left(t\right)-d_{b_{0}}\right)}+\sum_{s\in L_{B}}\frac{\alpha_{s}\left(f_{k}\right)}{\alpha_{b_{0}}\left(f_{k}\right)}e^{\frac{-j2\pi }{\lambda_{k}}\left(d_{s}-d_{b_{0}}\right)}+Z$$

It is now clearly seen from the equation above that the calibration preserves CSI and removes the phase rotation term though with a constant scaling in amplitude and phase shifting by $\alpha_{b_{0}}\left(f_{k}\right)$ and $d_{b_{0}}$ respectively. To validate the performance of the proposed CSI calibration model, an experiment involving Equation (8) was conducted in Section 4.

### 3.2. Motion Detection Analysis

**Figure 6:**
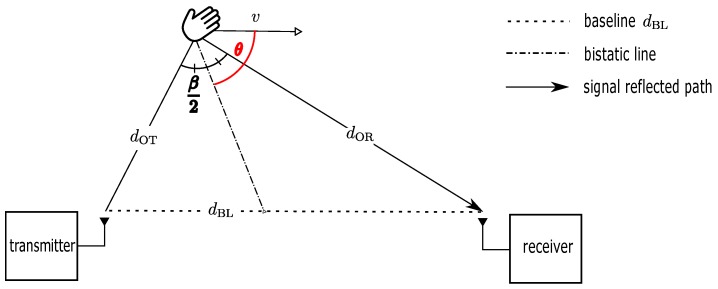
Geometry of bistatic Doppler radar according to [33].

To understand clearly how the presence of moving IOs influences signal propagation paths geometrically, this motion effect is explained in terms of the bistatic Doppler radar system. This radar system is commonly used for speed and position estimation of moving objects from the reflected RF signal between a transmitter and receiver separated by the length of baseline $d_{\mathrm{BL}}$. According to [33,41], the geometry of the bistatic radar in Figure 6 defined in the following will be derived to model the bistatic Doppler. The fixed transmitter, moving object, and fixed receiver forms a bistatic angle β. Therefore, if the object is moving with a constant speed *v* within a short period ∂t in the direction of θ with respect to the bistatic line, the bistatic Doppler frequency can be calculated as a function of the instantaneous change of path length,

(9) $$\begin{array}{cc} f_{d}=\frac{1}{\lambda }\frac{\partial }{\partial t}\left(d_{\mathrm{OT}}+d_{\mathrm{OR}}\right) & =\frac{1}{\lambda }\left[v\cos\left(\theta +\frac{\beta }{2}\right)+v\cos\left(\theta -\frac{\beta }{2}\right)\right]\\ & =\frac{2v}{\lambda }\cos\left(\theta \right)\cos\left(\frac{\beta }{2}\right) \end{array}$$

This equation evidently explains the effect of motion perceived by the reflected RF signal which depends not only the object’s speed and RF frequency but also the relative position with respect to the transmitter and receiver. The sign of Doppler frequency mostly depends on cos(θ) which results in the negative Doppler frequency when the angle is greater than $90^{\circ }$, and vice versa. Speed derived from the magnitude of $f_{d}$ is factored by θ and β, thus it should be smaller than the object speed. For instance, motion may not be detected when a the direction of motion is perpendicular to the bistatic line as the factor, cos(θ)=0, entirely eliminates $f_{d}$.

Bistatic Doppler model in Equation (9) could be used to analytically explain the rotation of CSI phase in the dynamic component due to moving IOs as shown in Equation (3a). According to [42], there are two types of Doppler-domain transfer functions where CSI can transform into. Doppler-variant transfer function $B(f_{d},f)$ represents the channel in the Doppler and frequency domains whereas Doppler-variant impulse response $s(f_{d},\tau )$, also known as spreading function, describes the channel in terms of delay τ and Doppler. Both functions can be calculated by taking the Fourier transform with respect to time *t* from H(t,f) and h(t,τ) respectively. Here h(t,τ) is the channel impulse response which can be calculated by taking the inverse Fourier transform of H(t,f) with respect to frequency.

Although using the Fourier transform allows the analysis of overall Doppler spectrum in $B(f_{d},f)$ and $s(f_{d},\tau )$, it entirely loses time information in the process. One may use the short-time Fourier transform to find the transient Doppler frequency but with a fixed time and Doppler frequency resolution due to the uncertainty principle. On the other hand, the Wavelet transform relaxes this constraint by varying the resolution at different frequency bands without violating the principle [43,44]. As a result, a better resolution of Doppler frequency (with low time resolution) at low frequencies can be achieved. This feature, however, is opposite in high frequencies with a higher time resolution but a poorer Doppler frequency resolution. Mathematically, the Wavelet transform is a convolution of a time-variant signal g(t) which is H(t,f) or h(t,τ) in our case and the conjugate of mother Wavelet function $\psi^{*}\left(t\right)$

(10) $$W\left(a,u\right)=\frac{1}{\sqrt{a}}\int_{-\infty }^{-\infty }g\left(t\right)\psi^{*}\left(\frac{t-u}{a}\right)dt,$$

where g(t) is represented in terms of wavelet coefficient W(a,u) at time *u* with scale *a* which is inversely proportional to the center frequency of $\psi (\frac{t-u}{a})$. In addition to the type of ψ(t), an analytic Wavelet function is necessary since CSI is a complex signal in order to analyze both amplitude and phase components. Therefore, we selected the analytic Morlet wavelet for our experiment as it was widely used for Doppler analysis [45,46,47].

## 4. Experiment and Evaluation

This section explains the details of the experimental setting and phase rotation mitigation performance of the CSI calibration method explained in Section 3.1. The capability to capture the bistatic Doppler frequency due to the motion of the calibrated CSI described in Section 3.2 was also experimented by using the Wavelet transform.

### 4.1. Experimental Setup

**Figure 7:**
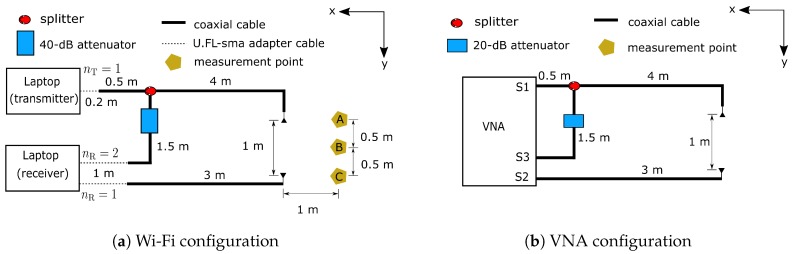
Physical configuration of the measurement scheme.

A testbed was implemented on commercial equipment. Two laptops equipped with Intel 5300 802.11n MIMO Wi-Fi NICs were used as Wi-Fi stations. Commercial Wi-Fi 6dBi-omnidirectional antennas operating in both 2.4 GHz and 5 GHz bands were externally connected to the Wi-Fi NICs through a coaxial cable with 0.77 velocity factor and U.FL-to-SMA adapter cables. A Mini-Circuits ZFSC-2-10G 2-way RF power splitter/combiner was applied at the first transmit antenna for dividing the signal into the b2b channel where a 40dB-attenuator was inserted at the cable connected to the second receive antenna. Figure 7a shows the connection configuration and specific cable lengths. Linux 802.11n CSI tool developed by [12] was installed on both laptops that run Ubuntu version 12.04.1 to extract CSI from Wi-Fi packets. To control transmission parameters such as transmission rate and the number of active antennas, a Dell Latitude D530 laptop was set as the transmitter operating in Injection mode while an Acer Travelmate 5760 laptop was the receiver operating in monitor mode. The distance between the first transmit antenna and the first receive antenna was 1 meter. In all measurements, 2500 Wi-Fi packets were sent every second over the 5.31 GHz frequency band (Wi-Fi channel number 62) and 40 MHz bandwidth. Due to the CSI tool configuration, it only captures CSI with 30 subcarriers equally sampled every four subcarriers from a total of 114 which follows the CSI grouping number 4 according to standard [23] covering 36.25 MHz bandwidth.

For comparison, an E5071B VNA developed by Keysight Technologies (formerly known as Agilent’s Electronic Measurement), Santa Rosa, California, USA, was used as the standard for evaluating the performance of CSI after calibrating phase rotation. The $S_{21}$ between ports S1 and S2 was assigned for the wireless channel while $S_{31}$ was set to the b2b channel between ports S1 and S3. All physical connections are the same as those in the Wi-Fi configuration except for the absence of U.FL -to-SMA adapter cables and the use of a 20dB-attenuator as shown in Figure 7b. The reason a higher attenuation was used in Wi-Fi was to avoid the saturation of the received signal due to the vast difference of received signal strength between the b2b and wireless channels. Instead of sending 30 subcarriers, we set the VNA to sweep and equally transmit 30-tone frequencies over the same channel and bandwidth as Wi-Fi. A custom-made visual basic for applications (VBA) script was implemented inside the VNA to automatically measure the wireless channel and b2b channel every 4 ms under 70 kHz IFBW setting, the fastest sampling time the VNA can handle given this configuration. The script configured a bus trigger in the single mode for initiation and termination of each measurement. All channel information captured from both instruments were later processed in MATLAB R2018a developed by MathWorks Inc., Natick, Massachusetts, USA.

### 4.2. Evaluation of the CSI Calibration Model

**Figure 8:**
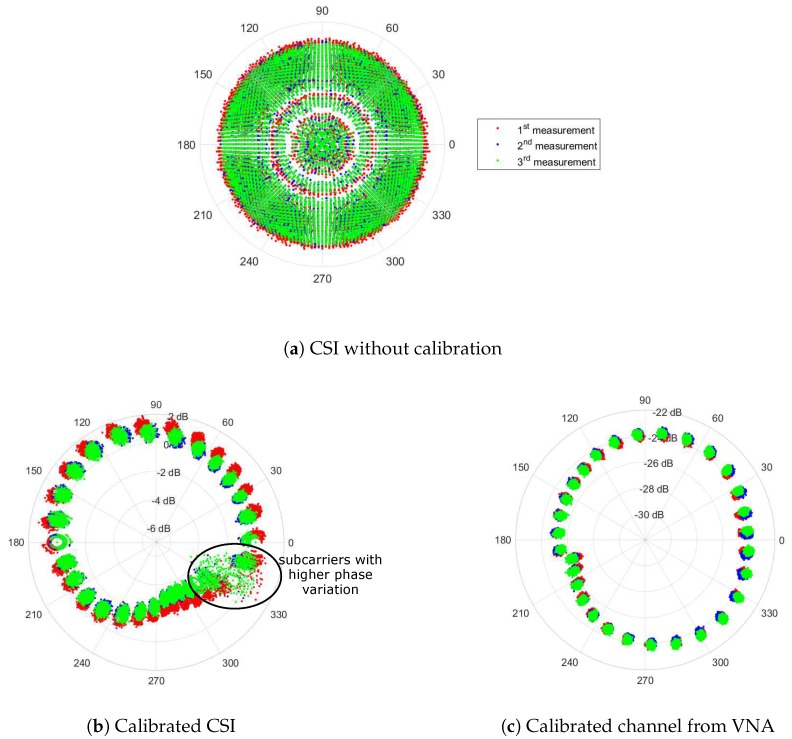
Polar plot of Wi-Fi CSI (**a**) before and (**b**) after calibration in comparison with (**c**) channel measured by VNA within a 10-second period.

The performance of our calibration method is determined based on how close the calibrated CSI to channel measured using the VNA which represented the CSI without phase rotations. Although there is virtually no phase rotation in VNA, the calibration method was also applied on the channel measured from the VNA for a fair comparison. In this experiment, measurement was conducted in the room without people (static environment) using both instruments within a 10-s period, and was repeated three times for reproducibility of the calibration. Figure 8a clearly depicted the effect of phase rotation in polar coordinate causing phase rotation similar to the b2b channel primarily tested in Figure 3. After the CSI calibration was performed, CSI phase became indistinctly more stable as shown in Figure 8b. The calibrated CSI has a similar trend to the VNA channel as depicted in Figure 8c with distinguishable 30 clusters in polar coordinates corresponding to subcarrier frequencies separated by a comparably constant phase shift except for a few subcarriers with a high amplitude and phase variation. It was found that the distorted subcarriers are those located near the bandwidth boundary which have fairly smaller path gain relative to other subcarriers. According to the CSI tool developer, this distortion is unavoidable due to a filter function applied during channel estimation. Nevertheless, ignoring the distorted subcarriers, standard deviation of CSI phase is acceptably low at about $1.64^{\circ }$ which is reasonably higher than the variation in the calibrated channel by the VNA.

**Figure 9:**
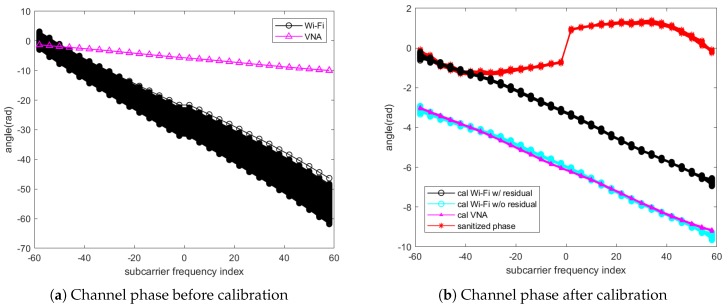
Comparison of phase of the calibrated CSI to the calibrated channel measured from VNA and phase sanitization method (**a**) before and (**b**) after calibration.

As it can be clearly observed in Figure 8b that the calibrated CSI at all three measurements produced the similar trend, only the result from the 2nd measurement was illustrated in Figure 9 to compare the performance of the calibrated CSI in a linear plot. In Figure 9a, the measured CSI phase in a black line with circle markers became unstable owing to the phase drifting away from the channel measured by the VNA depicted as the solid line with triangle markers. The effect of phase rotation and shift could also be explained by the instantaneous phase rotation model introduced in Equation (5). Besides the similarity of the calibrated CSI with the result by the VNA depicted as a black line with circle markers and a magenta line with small triangle markers respectively in Figure 9b, it also showed a constant phase offset around 3 radians relative to the calibrated channel measured by the VNA at all frequencies. After investigation, it was heuristically found that the additional offset was constantly changed every time the receiver was initialized. Hence we suspected that the residual PLO would be the cause of preventing the alignment with the absolute phase without calibrating all the channels. For the fair comparison, it is necessary to remove the residual offset from the calibrated CSI. As the residual offset is constant, we could be able to statistically calculate this offset by averaging the difference between the calibrated CSI and the ones from VNA. The calibrated CSI after removing the residual offset shown as a cyan line with circle markers in Figure 9b has virtually identical to the ones by the VNA at all frequencies with sightly small RMSE of 0.114, 0.123 and 0.116 radians which correspond to $6.50^{\circ }$, $7.04^{\circ }$, $6.62^{\circ }$ for three measurements respectively. By comparison with the existing methods [15,22,29,30], the result of CSI after applying the phase sanitization approach was plotted as the red line with asterisk markers in Figure 9b. The sanitized phase obviously had a totally different phase pattern to the result measured using the VNA because the sanitation removed the part of CSI phase together with the phase rotation.

**Figure 10:**
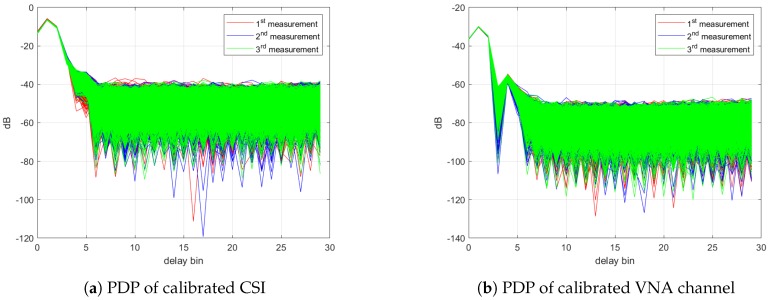
Power delay profile of (**a**) calibrated CSI and (**b**) calibrated VNA channel.

As it seems phase rotations are almost convincingly removed except for the residual PLO, it would be possible to compare the power delay profiles (PDP) of CSI which is simply the magnitude squared of channel impulse response. The Hanning window was multiplied with the channel before transformation to suppress the effect of the distorted subcarriers as well as the side lobes. As depicted in Figure 10, both PDPs of calibrated CSI and the ones from VNA have a single-peak PDP which is due to the narrowband channel, and the similarity between two profiles can be seen with clarity. Due to calibration, the power level of both profiles is the relative power with respect to the power of the b2b channel. As a result, this indicates the possibility of using the calibrated CSI for motion analysis at each delay bin separately from the channel impulse response. Although the LoS path (the strongest path) is only 1 m, the peak is located at the 2nd bin in both profiles which corresponds to a propagation delay of 27.58 ns because each delay bin was offset by the propagation path in the cable.

### 4.3. Motion Detection Analysis with Wavelet Transform

**Figure 11:**
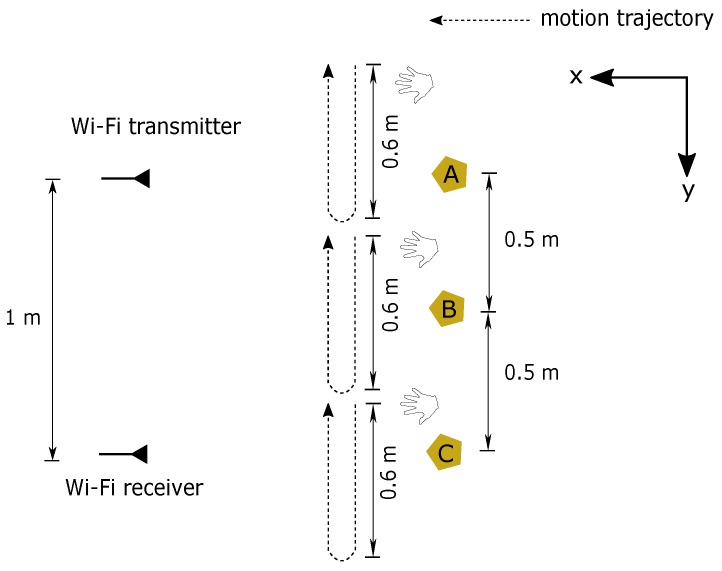
Experiment scenarios of hand motion detection at point A, B, and C.

In this experiment, simple hand gestures were used for motion detection analysis performed at three marked points depicted in Figure 7a while CSI was continuously estimated at the Wi-Fi receiver for 10 s in the room without other people. Before measurement began, a person stood facing in the x-direction and raised his hand up to the same level as the transmitter and receiver. During the measurement, his right hand was moved horizontally towards the y-direction for about 60 cm. After pausing for 2 s, his hand was pulled back (negative y-direction) to the original position as depicted in Figure 11. These gestures were performed twice in the single measurement. For analysis of bistatic Doppler, the channel impulse response at the delay bin with the highest power was chosen in this scenario. This is because both reflected path’s signal from the moving hand and the LoS path were within the same delay bin. Therefore, by limiting our analysis to only this single delay bin, we are effectively focusing only on the LoS path and the strong reflected path from the hand, thus the Doppler frequency should be more easily observed.

**Figure 12:**
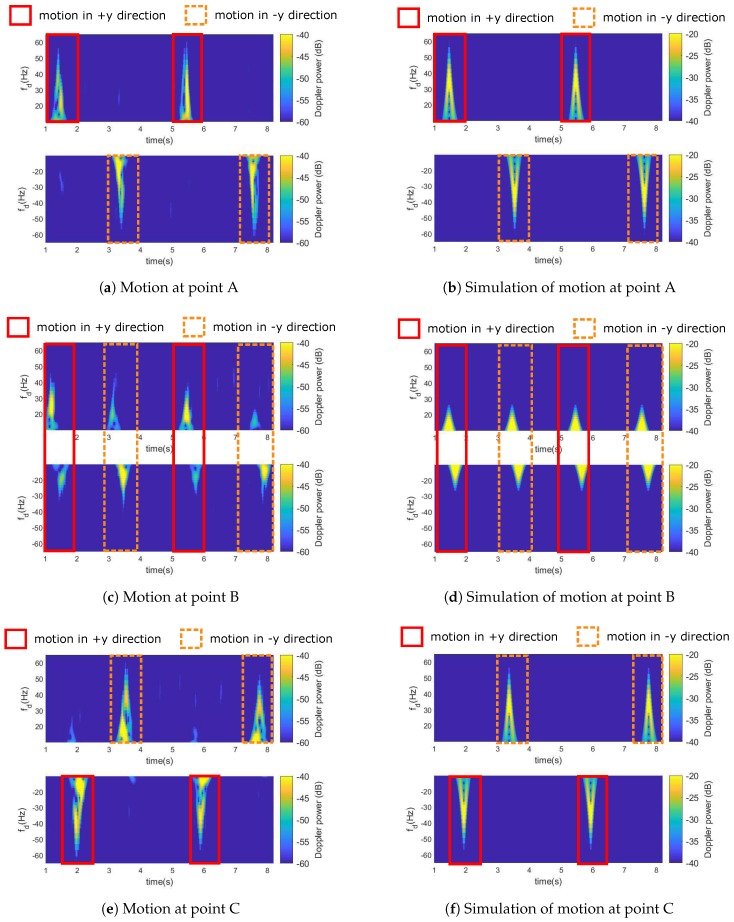
Bistatic Doppler spectrum produced by hand gestures at three different locations in the Wavelet time-frequency domain from the calibrated CSI measurement (**left**) and the simulation (**right**).

The measurement was repeated six times at each marked point for reproducibility. Since the results at the same point are similar, one of the measurements at positions A, B, and C were illustrated at the left side of Figure 12. It revealed the effect of motion where a stronger Doppler power spectrum represents bistatic Doppler frequency. In all cases, bistatic Doppler has a bell-shaped profile with a duration of around half a second on average. These gestures were similar to a speed profile of a wrist when handwriting as [48]. The highest bistatic Doppler frequency is about 60 Hz or roughly 1.7 m/s without factoring direction of motion parameter cos(θ). Although we were not able to calculate a precise motion trajectory from cos(θ) and cos(β/2) in this measurement, these figures can tell the relative direction from the sign of bistatic Doppler. For instance, Doppler frequency at point A had the positive sign when the hand was moving towards the y-direction and a negative sign when moving in the negative y-direction as shown in Figure 12a because the direction factor cos(θ) is less than and higher than $90^{\circ }$ respectively. Doppler sign was opposite at point C as shown in Figure 12e where it was negative in the y-direction and vice versa, and this can be explained by cos(θ) in the same manner. This effect also explained a sudden change of Doppler sign within a single gesture at point B as shown in Figure 12c from positive to negative in both y-direction and negative y-direction. Moreover, at point B, the magnitude of Doppler is comparatively smaller than others because of cos(θ)→0 in this measurement. The simulation of hand motion using the CSI model in Equation (3) and the bell-shaped arm movement model in [49] was conducted under the same configuration using in the measurement campaign. The Doppler frequency spectrum from the simulation depicted at the right side of Figure 12 could correctly predict the movement direction with corresponding to the sign of Doppler, and also showed similar Doppler profiles to the measurement at all marked positions.

This result could qualitatively confirm that the use of bistatic Doppler for motion analysis can be successfully achieved with calibrated CSI. Moreover, fine-grained or micro-motion detection may use this calibration model as a basis for further investigation. Although calibrated CSI does not remove the constant phase offsets from the residual PLO, it should not have any impact for motion analysis because any constant value will be added to zero Doppler frequency component during transformation. By comparison with related works, it would be difficult to produce correctly the Doppler frequency profiles as depicted in Figure 12 for the result from the phase sanitization and the phase difference approach because they do not have the original phase information. Using only CSI amplitude may still able to analyze motion but has the limitation to observe the relative direction of motions from the sign of bistatic Doppler frequency.

## 5. Conclusions

This paper first described how WI-Fi channel state information is estimated and the significance of spatial mapping and cyclic shift diversity to the additional shift of CSI phase which needs to be removed before further analysis. Secondly, a CSI calibration method was introduced to virtually remove time-varying phase rotation while preserving original phase information. The proposed technique uses a so-called reference CSI, a time-invariant channel that experiences the same phase rotation as the target CSI. Practically, a back-to-back channel representing a wired channel in the cable between transmit and receive antenna ports was selected as the reference CSI. Bistatic Doppler radar model was employed to describe the effect of motion as Doppler frequency in CSI.

An experiment was conducted to validate the performance of our method by comparison with a channel measured by a VNA. The result showed that phase rotation was mitigated according to our propose CSI calibration model and portrayed a similar pattern but with a higher phase variation than the channel from the VNA. Although a constant phase offset possibly due to the residual of the PLO still existed after calibration, it should have small significant for CSI-based motion analysis as any constant would result in zero Doppler frequency. For a fair validation, we statistically removed the constant phase offset from the calibrated CSI. Promisingly, the data produced high correlation to the result measured using the VNA with approximately 0.117 radians RMSE phase error. Another experiment was carried out to observe the effect of hand waving on the calibrated CSI at three different locations. The movement profile was visualized in the time-Doppler domain after the CSI was Wavelet transformed. A bell-shaped pattern of Doppler frequency caused by the hand gesture was observed where the sign of Doppler which indicates the motion direction was correctly predicted according to the bistatic Doppler model and also agreed with the simulation result. To the best of our knowledge, this CSI calibration model is the solution to practically obtain Wi-Fi CSI without phase offset and thus allowing full use of the Wi-Fi channel for further in-depth CSI-based motion analysis.
